# Capacity *in vacuo*: an audit of decision-making capacity assessments in a liaison psychiatry service

**DOI:** 10.1192/pb.bp.115.052613

**Published:** 2017-02

**Authors:** Benjamin W. J. Spencer, Gareth Wilson, Ewa Okon-Rocha, Gareth S. Owen, Charlotte Wilson Jones

**Affiliations:** 1 Department of Psychological Medicine, Institute of Psychiatry, Psychology and Neuroscience, King's College London; 2 South London and Maudsley NHS Foundation Trust; 3 Darent Valley Hospital, Dartford, Kent

## Abstract

**Aims and method** We aimed to audit the documentation of decision-making capacity (DMC) assessments by our liaison psychiatry service against the legal criteria set out in the Mental Capacity Act 2005. We audited 3 months split over a 2-year period occurring before, during and after an educational intervention to staff.

**Results** There were 21 assessments of DMC in month 1 (6.9% of all referrals), 27 (9.7%) in month 16, and 24 (6.6%) in month 21. Only during the intervention (month 16) did any meet our gold-standard (*n* = 2). Severity of consequences of the decision (odds ratio (OR) 24.4) and not agreeing to the intervention (OR = 21.8) were highly likely to result in lacking DMC.

**Clinical implications** Our audit demonstrated that DMC assessments were infrequent and poorly documented, with no effect of our legally focused educational intervention demonstrated. Our findings of factors associated with the outcome of the assessment of DMC confirm the anecdotal beliefs in this area. Clinicians and service leads need to carefully consider how to make the legal model of DMC more meaningful to clinicians when striving to improve documentation of DMC assessments.

All doctors are often required to make assessments of their patients' decision-making capacity (DMC). This role is prominent in liaison psychiatry services, as psychiatrists may be asked to provide a second opinion on DMC for other medical specialties (e.g. regarding consent for a surgical procedure). In the UK, psychiatric second opinion tends to be requested following refusal of treatment by a patient,^1^ but also when the assessment is likely to be highly complex or is driven by an underlying psychiatric disorder.^1,2^ DMC is also routinely assessed in the UK in psychiatric patients in emergency settings such as accident and emergency (A&E) departments. At the time of the audit guidance by the Care Quality Commission^3^ recommended that assessments regarding DMC to consent to treatment and admission should be made on all patients at the point of admission to a psychiatric hospital to clarify whether it was an admission authorised through the consent of a patient with DMC or under the ‘best interests’ procedures in a patient who is assenting but lacking DMC.

In England and Wales the legal criteria through which DMC is assessed are provided by the Mental Capacity Act 2005, with further guidance in its Code of Practice.^4^ Under the Act, for a person to lack DMC evidence is required that they suffer from ‘an impairment of, or a disturbance in the functioning of, the mind or brain’ (Section 2(1)), and that as a consequence of this they are unable to perform at least one of the following tasks: ‘(a) to understand the information relevant to the decision, (b) to retain that information, (c) to use or weigh that information as part of the process of making the decision, or (d) to communicate [their] decision’ (Section 3(1)). DMC is ‘decision specific’ rather than a ‘blanket’ or global ability: it is tailored to the specific decision at hand and recognises that different factors may influence different decisions.

There are additional considerations during the assessment of DMC, in that the assessor must also take heed of the principles of the Mental Capacity Act 2005 which include that ‘A person must be assumed to have capacity unless it is established that he lacks capacity’ and ‘A person is not to be treated as unable to make a decision unless all practicable steps to help him to do so have been taken without success’ (Section 1). Recently the House of Lords Select Committee on the Mental Capacity Act 2005 heavily criticised the implementation of the Act: ‘Our evidence suggests that capacity is not always assumed when it should be. Capacity assessments are not often carried out; when they are, the quality is often poor. […] The presumption of capacity, in particular, is widely misunderstood by those involved in care’.^5^

We know that patients lacking DMC to consent to treatment are common in both general medical in-patient (around 40%)^6^ and psychiatric in-patient settings (around 60%).^7^ An assessment and decision regarding DMC has significant repercussions for the patient, either respecting autonomy or paving the way to surrogate decision-making under ‘best interests’. It is therefore important to document clear justification for a decision reached, under the statutory criteria of the Mental Capacity Act 2005. Previous work in the USA has shown poor documentation of DMC in a retrospective study of in-patient lumbar punctures authorised by a surrogate decision-maker (3 patients had DMC documented out of 25 procedures authorised by a surrogate, although in 21 cases there was enough information to ‘infer’ lack of DMC).^8^

Our aim was to audit the documentation of DMC assessments provided by our liaison psychiatry service against the Mental Capacity Act 2005 and its Code of Practice, and the guidance issued by contemporary literature.^9^ Subsequently, we employed an educational intervention in our service to see whether this improved documentation of the assessments. We collected detailed information on the factors influencing the assessment of DMC to see whether this had an impact on the quality of DMC assessment or of the intervention.

## Method

All new referrals received by the King's College Hospital Liaison Psychiatry Team, South London and Maudsley NHS Foundation Trust (SLaM), during the month of November 2011 were collated. The team is split into the accident and emergency team and the general in-patient team covering all referrals. In working hours there are also specialist child and adolescent mental health services (CAMHS), older adults, neuropsychiatry and perinatal psychiatry teams. All referrals outside of working hours are taken on by the general service. CAMHS referrals were excluded from this audit.

Assessments of DMC performed by members of the team are completed without a formal template or form and were documented in the SLaM electronic medical record (EMR) as free text. All patient contacts by the team need to be recorded in the SLaM EMR.

The EMR was searched for documentation of assessments of DMC by searching for the word ‘capacity’ and any statement declaring that the patient had or lacked DMC was taken to be an assessment of DMC. The entire duration of the patient episode related to that referral was audited. Statements that suggested the patient may or may not have DMC but that did not make formal declaration were taken by this audit to be an assessment of DMC. The exception to this were statements that suggested the person may lack DMC, but that formal assessment could be delayed.

We set our gold-standard to require documentation of: (1) justification of the timing of the assessment/attempts to maximise DMC in accordance with the principles of the Mental Capacity Act 2005 and; (2) the full statutory criteria of DMC (presence or absence of a disorder of ‘mind or brain’, and performance on the four key abilities). We excluded the need for an explicit statement linking psychopathological features of the disorder of ‘mind or brain’ to any deficit found in the four key abilities given that some patients were found to not have a disorder of ‘mind or brain’.

Further information was gathered, including: basic demographic information, whether the assessment took place in or out of hours, the decision for which DMC is being assessed, the professional background of the assessor, and whether the person agreed or objected to the intervention/option proposed. The severity of potential consequences to the patient regarding refusal of the intervention for which DMC was assessed was also quantified, and classed as mild, medium and severe risk of adverse outcome (by a psychiatrist with experience of DMC assessments and patient management in this clinical environment). An example of a severe risk is a refusal of admission into a psychiatric hospital by a patient with florid psychosis or refusal of life-sustaining medical treatment; an example of mild risk is a refusal to contact relatives for information-sharing regarding the patient's clinical episode.

Following the audit in November 2011 (month 1), we analysed the initial results. Given that none had met our gold-standard (see below), we designed an educational intervention to improve clinicians' understanding of the legal framework of the assessment of DMC and how to document this appropriately. The intervention took place during the week psychiatrists in training (senior house officers (SHOs)) change jobs (February 2013, month 16), and continued for the following 4 weeks. It involved the lead auditor (B.S.) meeting with the medical (SHO) and nursing (psychiatric liaison nurses (PLNs)) members of the liaison team and explaining the gold-standard of DMC documentation; presenting this to the on-call SHOs during their induction as they may cover the team out of hours; and emailing the SHO cohort and the senior doctors on call (specialist registrars (SpRs)) on a weekly basis with the guidance. Posters with the guidance were put up in the departmental offices seen by the PLNs and SHOs.

The 4-week period in February 2013 (month 16) during which the intervention was applied was audited, along with the month of July 2013 (month 21), using the methods described above. This audit was reviewed and approved by the trust Psychological Medicine Audit Committee in January 2012.

## Results

In month 1, there were 21 (6.9%) assessments of DMC for 306 referrals, in month 16 there were 27 (9.7%) assessments for 278 referrals, and in month 21 there were 24 (6.6%) assessments for 365 referrals (Table 1). Overall, DMC was assessed in 72 (7.6%) out of a total of 949 referrals.

**Table 1:** Frequency of DMC assessments, demographics, and number meeting audit standards by month^a^

	Month 1	Month 16	Month 21	Total sample
Total referrals, *n*	306	278	365	949

DMC assessments, *n* (%)	21 (6.9)	27 (9.7)	24 (6.6)	72 (7.6)

Age, years: mean (s.d.)	45.2 (14.8)	39.3(15.4)	45.6 (15.0)	43.1 (15.2)

Female, *n* (%)	12 (57.1)	14 (51.9)	14 (58.3)	40 (55.6)

Patients found to have DMC, *n* (%)	6 (28.6)	16 (59.3)	14 (58.3)	36 (50)

Assessments documenting the statutory criteria, *n* (%)	2 (9.5)	6 (22.2)	2 (8.3)	10 (13.9)

Assessments meeting the audit gold-standard, *n* (%)	0	2 (7.4)	0	2 (2.8)

a. Some patients had more than one DMC assessment. There were no significant differences between months.

None met the gold-standard in months 1 or 21, however, 2 (7.4%) did during the intervention in month 16 (Table 1) and both of these assessments were performed by the SHOs who had received the educational intervention. Results were similar when using our lower standard of documentation of the full statutory criteria: *n* = 2 (9.5%) in month 1, *n* = 6 (22.2%) in month 16 and *n* = 2 (8.3%) in month 21. The majority of those failing this standard missed out several elements; only 3 (4.2%) assessments missed reaching the standard through missing only one of the four key abilities, whereas 38 (52.8%) assessments documented none of the four key abilities. We therefore cannot conclude the educational intervention had any impact at all.

Where recorded, we looked at how frequently the key abilities to be tested were lacking in people who lacked DMC. Lacking the ability to ‘use or weigh’ information was most common (*n* = 19, 73.1% where recorded), followed by ‘understanding’ (*n* = 10, 43.5% where recorded), ‘retaining’ (*n* = 8, 57.1% where recorded) and ‘communication’ (*n* = 4, 33.3% where recorded).

The majority of assessments of DMC were performed by doctors (*n* = 51 (70.8%) *v. n* = 21 (29.2%) assessments performed by the PLNs). Of the PLNs' assessments only 3 (14.3%) patients were found to lack DMC, whereas of the doctors' assessments 33 (64.7%) patients lacked DMC (Table 2). PLN assessment was significantly more likely to result in a positive DMC than a doctor assessment (OR = 11.0, 95% CI 2.9 to 42.5). The doctors met the gold-standard (*n* = 2, 4%) and full statutory criteria (*n* = 9, 18%) more often than the PLNs (*n* = 0 and *n* = 1, 5% respectively).

**Table 2:** Outcome of DMC assessment based on assessing clinician

	DMC present		
	No	Yes	Total
Assessing clinician, *n* (%)			
PLN	3 (14)	18 (86)	21 (29)
Doctor	33 (65)	18 (35)	51 (71)

Total, *n*	36	36	72

DMC, decision-making capacity; PLN, psychiatric liaison nurse.

We separated the types of decisions to be made by the patient into those that involved ‘psychiatric admission or treatment’ and ‘medical admission or treatment’. For the purposes of the audit, decisions to start a new admission in hospital or discharge oneself from a current admission were seen as interchangeable. Medical and psychiatric decisions were not mutually exclusive and a proportion of patients were assessed for both. There were assessments of DMC that did not focus on these decisions, but they were a minority and focused on decisions not normally tested in this setting, such as DMC to make a decision around ongoing homelessness (*n* = 1) and ongoing abusive relationship/domestic abuse (*n* = 2). These non-treatment-focused assessments of DMC all occurred as part of the psychiatric assessment by a PLN or doctor rather than following a request for second opinion from the medical teams.

We found that the proportion of assessments of DMC for medical admission or treatment formed the majority of assessment at the start of the audit month 1 (*n* = 17, 81%), but this reduced over the course of the audit in month 16 (*n* = 12, 44%) and month 21 (*n* = 8, 33%; Pearson's χ^2^ = 9.91, *P* = 0.007) (Table 3). Conversely, assessments for psychiatric admission or treatment were the minority at the start of the audit (*n* = 5, 24%) and increased in month 16 (*n* = 13, 48%) and month 21 (*n* = 13, 54%), although the differences were not statistically significant.

**Table 3:** Decisions for which DMC was assessed and numbers agreeing with the intervention by month

	Month 1	Month 16	Month 21	Total sample *n* = 72
Decisions to be made, *n* (%)				
Medical admission or treatment	17 (81)^a^	12 (44)^a^	8 (33)^a^	37 (51)
Psychiatric admission or treatment	5 (24)	13 (48)	13 (54)	31 (43)

Agreement status, *n* (%)				
Agreeing	5 (24)	15 (56)	12 (50)	32 (44)
Not agreeing (or unable to express a choice/not documented)	16 (76)	12 (44)	12 (50)	40 (56)

DMC, decision-making capacity.

a. Pearson's χ^2^ = 9.91, *P* = 0.007.

We separated the choices of people who were having their DMC assessed into agreeing with the intervention proposed by the assessor/medical team and objecting/unable to express a choice/unknown. Fewer people were assessed who agreed with the intervention in month 1 (*n* = 5, 24%), but in months 16 and 21 they made up half of those assessed (Table 3). Agreement with the intervention was strongly associated with a finding of DMC: 26 (81%) of those agreeing with the intervention were found to have DMC, compared with only 10 (25%) of those who did not agree (either objecting or otherwise) (Table 4). This was highly statistically significant (Pearson's χ^2^ = 22.50, *P*<0.001). Most assessments made by the PLNs were done in patients agreeing to the intervention (*n* = 15, 71%), contrary to doctor assessments (*n* = 17, 33%).

**Table 4:** Outcome of DMC assessment based on agreement with the proposed intervention^a^

	DMC present		
	No	Yes	Total
Agreement status, *n* (%)			
Agreeing	6 (19)	26 (81)	32 (44)
Not-agreeing (or unable to express a choice/not documented)	30 (75)	10 (25)	40 (56)

Total, *n*	36	36	72

DMC, decision-making capacity.

a. Pearson's χ^2^ = 22.50, *P*<0.001.

A logistic regression was performed to ascertain the effects of the assessor (PLN or doctor), agreement with the intervention and consequences of the decision. Initial models also included decision to be made and underlying mental disorder, however, these were removed from the final model as they had no effect.

The final regression model was statistically significant (Pearson's χ^2^ = 45.81, *P*<0.001). The model explained 64.7% of the variance (Nagelkerke R^2^) and correctly classified 81.2% of the outcome of the assessments of DMC.

Factors associated with the finding of lack of DMC were: more severe consequences of the decision (OR = 24.4, 95% CI 3.47 to 171.8), not agreeing with the intervention (OR = 21.8, 95% CI 4.0 to 118.8), and assessment by doctor rather than PLN (OR = 14.9, 95% CI 2.1 to 104.5).

## Discussion

We have shown evidence that documentation of 72 DMC assessments in 3 sampling months in a liaison setting was poor, with only 2 assessments reaching our gold-standard. The impact of a legal education intervention was very limited and was not sustained beyond the month in which it was applied.

There are several possible reasons as to why the proportion of assessments meeting our gold-standard was so low, even after the educational intervention. Clearly, a lack of documentation of all components of the assessment of DMC does not necessarily equate to these components not having been considered by the clinician assessing DMC. However, there is limited documentary justification of the nature of the clinical assessment and the legal model of DMC. Perhaps elements of the education intervention itself (design, style, length etc.) may not have been an effective means of conveying the information, although our audit was not designed to evaluate this. The explicit reference to the principles of the Mental Capacity Act 2005 in our gold-standard might have set the standard too high, but even our more lenient ‘full statutory criteria standard’ was only achieved in 13.9% of assessments during the course of the audit.

Where lies the difficulty in translation of the legal model to clinical assessments? In situations where evidence is presented to the Court of Protection (the civil court in England and Wales with the jurisdiction for cases in which an individual lacks DMC), the Court requires completion of prescribed forms that demand a level of evidence similar to our gold-standard. In a busy clinical environment it is easy to see how documentation of the presence or absence of DMC could be considered to be sufficient by clinicians. A process that might slow the system down (or be perceived as such) can be expected to be powerfully resisted.

It is interesting that the assessments of DMC by PLNs result more often in the patient being concluded to have decision-making capacity. This finding needs to be approached with caution given that DMC assessments were triggered by several different reasons in our audit, including either: (1) a second opinion assessment of DMC, usually in the context of a patient refusing treatment, in which the assessment would be performed by a doctor; or (2) an assessment of DMC in the context of admission to psychiatric hospital performed by any clinician.

As doctors performed all second opinions of DMC assessment, usually in the context of a patient refusing treatment, and they assess all patients who will need compulsory admission to hospital, there is a referral bias. The majority of DMC assessments performed by the PLNs were in the context of a patient agreeing with the suggested intervention, and hence were used to support the clinical assessment. If there is no dispute around the intervention offered, then DMC assessment has little consequence and it is easier to presume DMC.

The strong association between lack of DMC, high severity of outcome, refusal and lack of assent is striking. To our knowledge this is the first piece of work that has clearly demonstrated this association in real clinical practice. It would seem to suggest that clinicians when assessing DMC in practice use an outcome test of DMC rather than the functional test the law requires. Kim *et al*^10^ have shown that assessments of DMC by clinicians using video simulations of consent discussions around involvement in research are risk sensitive. This echoes early work on conceptualising DMC as necessarily risk sensitive.^2^ Owen *et al*^11^ reported an association between treatment refusal and DMC assessed using the MacArthur Competency Assessment Tool for Treatment. Although the association is striking, we consider it to be largely expected, given the selection bias that assessments of DMC performed as a second opinion by our service are normally prompted by treatment refusal in the context of a possible mental disorder, when refusal is likely to result in significant harm to the patient. It is reassuring that there are a proportion of assessments where people are found to have DMC despite the refusal and high severity of consequences, and we submit that this is evidence of careful clinical consideration of each case on its own merits.

Our audit has shown that there are limitations in the recording of assessments of DMC, and that uptake of an educational intervention was limited. We consider that this is likely due to the perceived disconnect between the legal assessment and clinical assessment. We would recommend that the next step in the audit cycle should include an educational intervention on the assessment of DMC with a formal evaluation, with exploration and focus on clinical factors and their relationship to legal criteria in order to be more acceptable for clinicians.

In conclusion, we have found evidence for the anecdotal belief on the impact of severity of consequences and agreement status of the patient on the outcome on their assessment of DMC. Reassuringly, these factors were not totally deterministic of the outcome but they do suggest that, in practice, the functional test of DMC is yet to fully bed down.
